# Development of microRNA-21 mimic nanocarriers for the treatment of cutaneous wounds

**DOI:** 10.7150/thno.39870

**Published:** 2020-02-10

**Authors:** Sun Young Wang, Hyosuk Kim, Gijung Kwak, Sung Duk Jo, Daeho Cho, Yoosoo Yang, Ick Chan Kwon, Ji Hoon Jeong, Sun Hwa Kim

**Affiliations:** 1 Center for Theragnosis, Biomedical Research Institute, Korea Institute of Science and Technology (KIST), Seoul 02792, Republic of Korea.; 2 KU-KIST Graduate School of Converging Science and Technology, Korea University, Seoul 02841, Republic of Korea.; 3 Nano-Bio Resources Center, Korea University, Seoul 02841, Republic of Korea.; 4 School of Pharmacy, Sungkyunkwan University, Suwon 440-746, Republic of Korea.

**Keywords:** microRNA-21, facial amphipathic bile acid, wound healing, gene therapy

## Abstract

**Rationale**: Of the regulatory microRNAs expressed in the wounded skin, microRNA-21 (miR21) plays a pivotal role in wound repair by stimulating re-epithelialization, an essential feature to facilitate healing and reduce scar formation. Despite their crucial roles in wound healing, synthetic exogenous microRNAs have limited applications owing to the lack of an appropriate delivery system. Herein, we designed an miR21 mimic nanocarrier system using facial amphipathic bile acid-conjugated polyethyleneimines (BA-PEI) for the intracellular and transdermal delivery of synthetic miR21 molecules to accelerate wound repair.

**Methods**: To design miR21 mimic nanocarriers, BA-conjugated PEIs prepared from three different types of BA at molar feed ratios of 1 and 3 were synthesized. The intracellular uptake efficiency of synthetic miR21 mimics was studied using confocal laser scanning microscopy and flow cytometry analysis. The optimized miR21/BA nanocarrier system was used to evaluate the wound healing effects induced by miR21 mimics in human HaCaT keratinocytes *in vitro* and a murine excisional acute wound model *in vivo*.

**Results**: The cell uptake efficiency of miR21 complexed with BA-conjugated PEI was dramatically higher than that of miR21 complexed with PEI alone. Deoxycholic acid (DA)-modified PEI at a molar feed ratio of 3:1 (DA3-PEI) showed the highest transfection efficiency for miR21 without any increase in toxicity. After effective transdermal and intracellular delivery of miR21/DA3 nanocarriers, miR21 mimics promoted cell migration and proliferation through the post-transcriptional regulation of programmed cell death protein 4 (PDCD4) and matrix metalloproteinases. Thus, miR21 mimic nanocarriers improved both the rate and quality of wound healing, as evident from enhanced collagen synthesis and accelerated wound re-epithelialization.

**Conclusion**: Our miRNA nanocarrier systems developed using DA3-PEI conjugates may be potentially useful for the delivery of synthetic exogenous miRNAs in various fields.

## Introduction

Acute wound repair is a physiological process orchestrated by a variety of cell types and matrix components with the aim to re-establish the integrity of the damaged tissue. This highly synchronized process is divided into three major stages, namely, the inflammatory, proliferative, and remodeling stage. Several microRNAs (miRNAs) have been shown to be potentially involved in the overall wound healing process in a highly decisive and coordinated manner 1-5. For instance, miR-130a, miR-132, miR-155, miR-198, miR-21, miR-31, and miR-378a are the most abundant miRNAs exhibiting different roles during wound healing 1, 6-9. Among the regulatory miRNAs expressed in the wounded skin, microRNA-21 (miR21) is known to exert crucial functions during wound repair mainly by regulating inflammation and proliferation, the two essential features for optimum healing and scar formation reduction 10-17. In the inflammatory phase, miR21 inhibits pro-inflammatory signals and discontinues the prolonged inflammatory phase, which may lead to chronic wounding 18-21. In particular, miR21 downregulates Toll-like receptor 4 (TLR-4)-mediated inflammation through the inhibition of the expression of programmed cell death protein 4 (PDCD4) 22, 23. Immediately after the inflammatory phase, the proliferation phase is initiated with fibroblast recruitment, collagen deposition, keratinocyte differentiation, and epithelialization 24. In particular, miR21 plays a pivotal role in inducing keratinocyte migration and epithelialization by regulating the expression of tissue inhibitors of metalloproteinases 3 (TIMP3) and T cell lymphoma invasion and metastasis 1 (TIAM1) 25.

**Figure 1:**
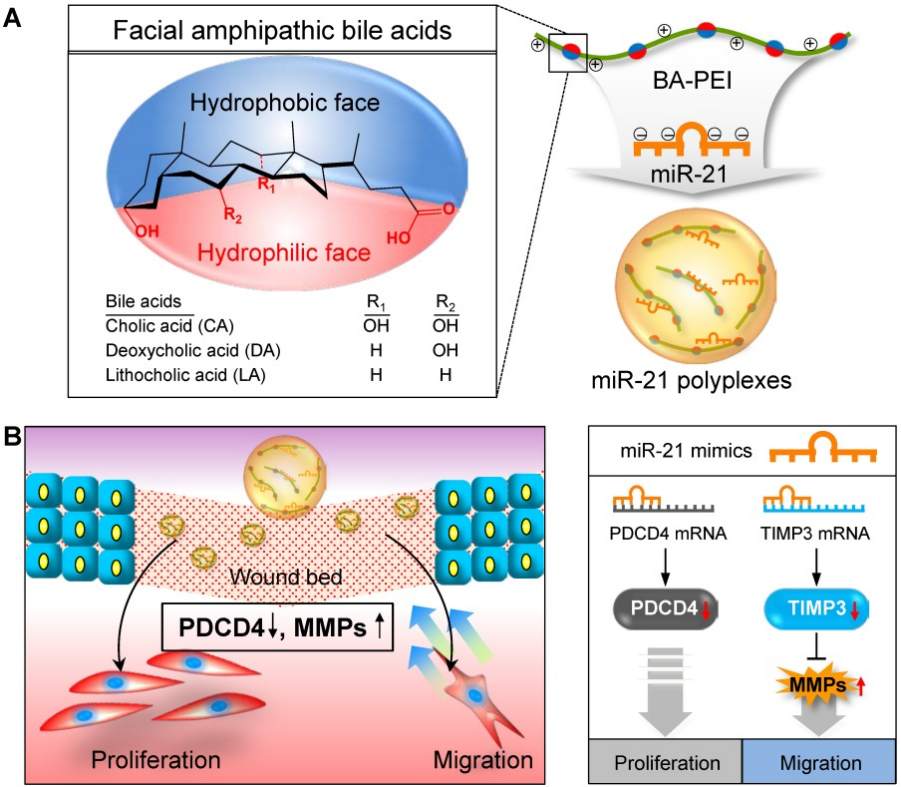
Schematic illustration of microRNA-21 (miR21) mimic nanocarriers for the treatment of cutaneous wounds. (A) Three types of bile acid-conjugated polyethyleneimine (BA-PEI) conjugates, including cholic acid (CA), deoxycholic acid (DA), and lithocholic acid (LA), were used to form polyplexes with miR21. (B) The effects of miR21 mimics in wound repair through the activation of cell proliferation and migration.

In this regard, synthetic miR21 mimics have recently gained attention in potential wound care therapies to promote rapid and complete healing. Significant advances have been made to improve our understanding about the mechanism underlying miRNA-mediated gene regulation. However, synthetic exogenous miRNA mimic or inhibitor-based therapies are severely limited owing to the lack of an efficient delivery system to achieve proper therapeutic effects 26. In general, exogenous miRNA therapeutics suffer from several limitations, including poor stability, low cellular uptake, and limited target tissue selectivity 27, 28. To improve the local delivery of miR21 and enhance the wound healing efficiency, here we introduced miR21 nanocarriers electrostatically complexed with three types of bile acid-attached polyethyleneimine (BA-PEI) conjugates, including cholic acid (CA), deoxycholic acid (DA), and lithocholic acid (LA) (**Figure 1A**). BAs are facially amphipathic end products of cholesterol metabolism comprising a rigid hydrophobic backbone with varying hydrophilic hydroxyl groups and flexible acidic side chains 29, 30. Nucleic acid molecules such as plasmid DNA and small-interfering RNAs (siRNAs) form electrolyte complexes (polyplexes) with BA-PEI; the unique facial amphiphilic structure of BA imparts good membrane permeability to polyplexes by membrane fusion, lipid structure rearrangement, and pore formation 31. BA-PEI was shown to enhance the delivery of several agents, including vascular endothelial growth factor (VEGF) gene and matrix metalloproteinase 2 (MMP2) siRNA 32, 33. Herein, we screen an optimum miRNA nanocarrier from a library of DA-PEI conjugates for the successful transdermal and intracellular delivery of miR21 mimics. This nanocarrier was found to post-transcriptionally regulate PDCD4 and MMPs to promote cell migration and proliferation and subsequently contribute to the acceleration of wound repair (**Figure 1B**).

## Materials and Methods

### Materials

An amine-modified negative control miRNA (sequence: 5′-GCG TAT TAT AGC CGA TTA ACG A-3′-amine) and a Cy5.5-labeled miR21 (sequence: 5′-UAG CUU AUC AGA CUG AUG UUG A-3′) were purchased from IDT (Coralville, IA, USA). mirVana® hsa-miR21-5p mimic (MC10206), cholic acid, deoxycholic acid, lithocholic acid, low molecular weight PEI (M_w_ 1800), and Cell Counting Kit-8 (CCK-8) were obtained from Sigma-Aldrich (St. Louis, MO, USA), while YOYO-1 iodide was supplied by Invitrogen (Carlsbad, CA, USA). NucleoSpin® miRNA was procured from Macherey-Nagel (Düren, Germany), and Mir-X miRNA First Strand Synthesis and SYBR qRT-PCR Kit, from Takara Bio (Shiga, Japan). The antibodies used in this study were as follows: β-actin (Abcam, ab227387, 1:2000), TIMP3 (CST, #5673S, 1:200), MMP2 (CST, #87809S, 1:200), and PDCD4 (Santa Cruz Biotechnology, SC-376430, 1:200).

### Cell culture

HaCaT human epidermal keratinocytes were maintained in Dulbecco's modified Eagle's medium (DMEM) supplemented with 10% fetal bovine serum (FBS) and 1% antibiotic-antimycotic (AA) at 37°C and 5% CO_2_. HaCaT cells were generally passaged after reaching 70% confluency by washing with phosphate-buffered saline (PBS) at pH 7.4 and harvesting with 0.5% trypsin-EDTA. Culture products mentioned above were obtained from Welgene Inc (Daegu, Korea).

### Synthesis of BA-PEI conjugates and preparation of miR21/BA polyplexes

The terminal amine groups of PEI and the carboxyl groups at the C-24 position of three BAs (CA, DA, and LA) were conjugated as described in previous studies 34. Each BA (1g, 2.5 mmol) was dissolved in methylene chloride and activated with dicyclohexylcarbodiimide (DCC) and N-hydroxysuccinimide (NHS) (stoichiometric molar ratio of DA/DCC/NHS = 1:3:3) at room temperature. Activated BA was added to 1.8 kDa PEI in methylene chloride at BA/PEI molar feed ratios of 1:1 and 3:1. The synthesized product was dried with a rotary evaporator and precipitated with cold acetone/ether (1:3 v/v %) solvent. The resulting products were dried with dry nitrogen, re-dissolved in deionized water, filtered, and lyophilized. For the complexation of miR21 and BA-PEI polyplexes, BA-PEI polymers were prepared in deionized water. A known amount of miR21 was simply added to the BA-PEI solution and the mixture was incubated for 30 min at room temperature. The BA-PEI complex was loaded on 2% agarose gel, pre-stained with Gel-Red, and electrophorized at 90 V for 30 min. Complex formation was visualized under UV irradiation.

### Cellular uptake of miR21/BA polyplexes

The cellular uptake of miR21 polyplexes was evaluated with flow cytometry and fluorescence microscopy. HaCaT cells were plated in 60 mm culture dishes at a density of 8 × 10^5^ cells and 35 mm glass bottom dishes at a density of 2×10^5^ cells for flow cytometry and fluorescence imaging, respectively. All dishes were incubated overnight to allow formation of cell monolayers. Prior to transfection, the seeded cells were washed with saline and starved for 4 h in serum-free DMEM. Cells were then transfected with miR21 polyplexes equivalent to 100 nM of miR21 in serum-free DMEM for 4 h. For fluorescence imaging, cells were fixed with 4% paraformaldehyde (PFA) for 10 min and counterstained with 4′, 6-diamidino-2-phenylindole (DAPI), followed by visualization with a laser scanning confocal microscopy (LSCM; LSM 510 Meta, Carl Zeiss Inc., Thornwood, NY). For flow cytometry, cells were harvested with 0.5% trypsin-ethylenediaminetetraacetic acid (EDTA) and suspended in cold PBS. Guava® easyCyte Flow Cytometer (Billerica, MA, USA) was used for analysis. To visualize the release of miR21 from polyplexes, miR21 was labeled with YOYO1-intercalating dye. Both miR21 and dye were mixed following the manufacturer's protocol. After labeling, the YOYO1-labeled miR21 and Cy5.5-labeled DA3 were complexed and incubated for 30 min at room temperature. Confocal fluorescence images were obtained as described above.

### Cytotoxicity, cell proliferation, and migration assays

Viable cells were counted with CCK-8 kit to determine the cytotoxicity of BAs and evaluate cell proliferation. HaCaT cells were seeded in 96-well plates at a density of 5000 cells/well. For both cytotoxicity and proliferation assays, cells were treated with miR21 polyplexes for 4 h and incubated in culture medium for 24 h. Assays were performed as per the manufacturer's protocol, and the absorbance was measured at 450 nm wavelength using VERSAmax^TM^ microplate reader (Sunnyvale, CA, USA). For wound scratch migration assay, HaCaT cells were seeded in six-well plates (SPL Life Sciences, Gyeonggi-do, Korea) at a density of 2 × 10^5^ cells/well and incubated at 37°C and 5% CO_2_ overnight to allow formation of a monolayer at 70% confluence. Cells were treated with saline, 50 nM of free miR-21, or miR21/BA polyplexes equivalent to 50 nM miR21 for 4 h. The monolayers were scratched with a 200 μL pipette tip, carefully washed with PBS, and cultured in serum-free medium. After 24 h incubation, cells were visualized under a culture microscope (Olympus CK40, Tokyo, Japan), and cell migration was evaluated by measuring the scored area with ImageJ software.

### Gene expression analyses

For real-time quantitative polymerase chain reaction (PCR), HaCaT cells were seeded in 60 mm cell culture dishes at a density of 2 × 10^5^ cells/dish. After 24 h treatment, miRNA was isolated using NucleoSpin® miRNA following the manufacturer's instructions. cDNA synthesis and real-time quantitative PCR were performed with Mir-X miRNA First Strand Synthesis and SYBR qRT-PCR Kit using miR21 primers (Genolution, Seoul, Korea) or the U6 primer. The thermal cycling conditions and calculation of relative copy numbers were carried out as suggested in the manufacturer's instruction. HaCaT cells were prepared for western blot analysis in a manner similar to that for real-time PCR. After 48 h, cells were lysed with a radioimmunoprecipitation assay (RIPA) lysis buffer supplemented with 1% protease inhibitor (Thermo Scientific, USA). The protein concentration of lysates was quantified with bicinchoninic acid (BCA) protein assay. Samples were mixed with a sodium dodecyl sulfate (SDS) gel-loading buffer and fractionated on 10% SDS-polyacrylamide gel electrophoresis gels. The separated protein bands were transferred onto polyvinylidene difluoride (PVDF) membranes, which were then incubated with primary and secondary antibodies specific for β-actin, MMP2, TIMP3, and PDCD4. Protein signals were detected using the SuperSignal™ West Femto Maximum Sensitivity Substrate (Rockford, IL, USA).

### Visualization of MMP2 activity *in vitro*

For the analysis of MMP2 activity, Cy5.5-Gly-Pro-Leu-Gly-Val-Arg-Gly-Lys(BHQ3)-Gly-Gly-OH peptide was synthesized with standard solid-phase Fmoc peptide chemistry as described in our previous work 34. HaCaT cells were seeded in 35 mm glass bottom dishes at a density of 2 × 10^5^ cells/dish. After 2 h of starvation, cells were treated with miR21/BA polyplexes equivalent to 50 nM miR21 for 4 h and incubated overnight in fresh culture medium. Prior to the use of the probe, lyophilized probe samples were dispersed in saline buffer. Cells were treated with MMP2 probe at a concentration of 10 μg/mL for 2 h, fixed with 4% PFA, and counterstained with DAPI. Fixed cells were observed with LSCM.

### *In vivo* skin excisional wound healing

Balb/c mice were purchased from Nara Biotech (Seoul, Korea). All animal experimental procedures were approved by the Institutional Animal Care and Use Committee (IACUC, 2017-086) at the Korea Institute of Science and Technology. A mouse excisional wound splinting model was generated as previously described 35. In brief, 5-week-old male Balb/c mice were anesthetized, and their hair from the dorsum removed. Two full-thickness excisional wounds were created on the dorsal surface with 6 and 8 mm disposable biopsy punches (Integra Miltex, York, PA, USA), and treated with saline, scramble-miR/DA3 (Scr-miR/DA3), miR21/DA3, and inhibitor-miR21/DA3 (Inh-miR21/DA3) equivalent to 2.5 μM miR21 on day 0 and 4 of injury. About 60 μL of samples were injected into the dermis at four sites around the wound, and additional 20 μL of samples were topically applied onto the wound bed. A 0.5 mm thick donut-shaped silicon splinting ring was secured around the wound with Scotch SuperGlue (3M, St Paul, MN, USA). To prevent infection, wounded sites were completely covered with Tegaderm (3M Health Care, St. Paul, MN, USA) and dressed with a self-adhering elastic bandage (Coban; Johnson & Johnson, Arlington, TX, USA). On days 0, 4, 8, 12, 16, and 20 of treatment, the wounds were cleaned and photographed with a digital camera. For dose-response experiment, saline or miR21/DA3 polyplexes equivalent to 1 and 5 μM miR21 were used for mouse treatment on day 0. For comparison of therapeutic effects of miR21 with recombinant human epidermal growth factor (rhEGF), mice were treated with 0.5 g nepidermin (Daewoong Pharmaceutical, Seoul, Korea) and 2.5 μM miR21 on day 0. For the analysis of protein expression level in tissues, mice were sacrificed and the skin around the wounds was harvested on day 12 of treatment. Tissue samples were transferred to RIPA buffer supplemented with 1% protease inhibitor and homogenized using a WiseMix homogenizer (Daihan Scientific, Seoul, Korea). Lysates were centrifuged for 20 min at 12,000 rpm at 4°C to remove any insoluble debris. Protein samples were quantified with BCA protein assay kit and prepared by adding SDS-gel loading buffer. Western blot analysis was performed as described above.

### Histological assessment

After 16 days from treatment, the wound bed was excised from the dorsal surface and fixed overnight at 4°C in PFA. The fixed samples were dehydrated and embedded in paraffin. After tissue embedding, a series of adjacent 6-μm-thick sections were cut, deparaffinized, and hydrated with xylene and ethanol solutions. The tissue slides were stained with hematoxylin-eosin (H&E).

### Visualization of MMP2 activity with MMP2 probe *in vivo*

After 5 and 10 days from treatment, mice were anesthetized and treated with lyophilized MMP2 probe dispersed in PBS at a concentration of 1 mg/mL at 4°C. Dorsal surface was cleaned with 70% (v/v) ethanol, and 30 μL of MMP2 probe solution was topically applied onto the wound bed. The wounds were dressed with Tegaderm and Coban bandage. After 24 h, all dressings were removed and the wounded site was washed with saline and 70% (v/v) ethanol to remove any remaining MMP2 probe and debris. Fluorescence imaging was performed to visualize MMP2 signal around the wounded site using IVIS® II Lumina (Caliper Life Sciences, Hopkinton, MA).

### Statistical analysis

All data are expressed as mean ± standard deviation (SD) and analyzed using Origin pro 8 software package (OriginLab Corp, MA, USA). All groups were compared with the Student *t*-test. A value of *p* < 0.05 was considered statistically significant.

## Results and Discussion

### Selection of BA-PEI polymers for miR21 nanocarrier preparation

**Figure 2:**
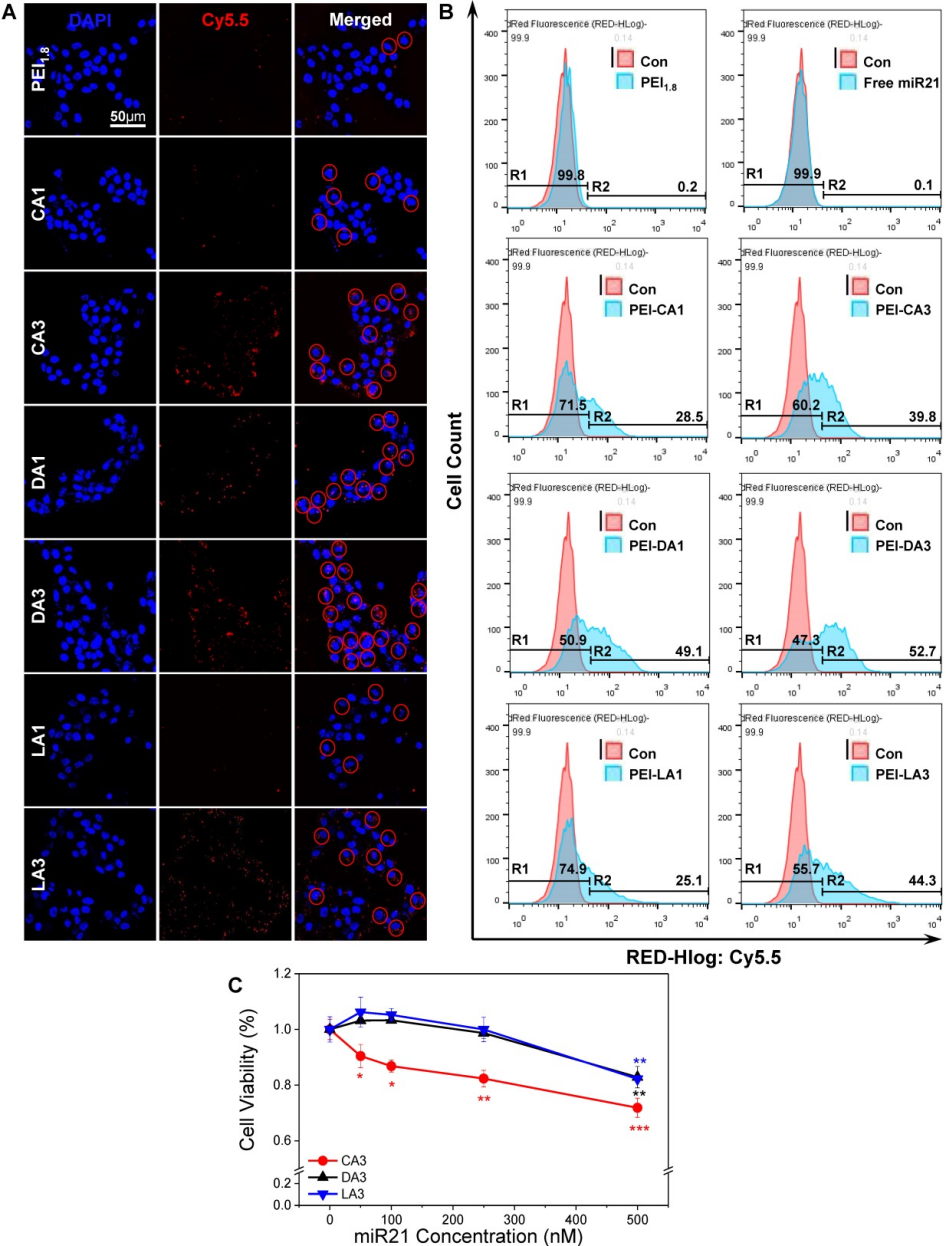
Selection of bile acids to form miR21 complexes. (A) Representative fluorescence microscopy image after transfection of Cy5.5-conjugated miR21/BA polyplexes. (B) Flow cytometry analysis showing the transfection efficiency of BA-PEI. (C) Cytotoxicity of CA3, DA3, and LA3-PEI carriers. Data are mean ± *SD* (*n* = 6); *p < 0.05, **p < 0.01, ***p < 0.001 versus 0 nM miR21.

Before assessing the wound healing effects of miR21 nanocarriers, their ability to delivery miR21 mimics was evaluated in HaCaT keratinocyte cells. BA polymer conjugates were expected to neutralize the strong negative charge of nucleic acids, the factor that limits the intracellular delivery of miRNAs 36. Although the mechanism of action is unclear, BA moieties may serve as cell-penetrating peptides (CPPs) to mediate the transfer of macromolecules across the cell membrane via endocytosis-independent and endocytosis-dependent processes and destabilization of the plasma membrane 37. In addition, BAs have been used for the formulation of new therapeutic systems to deliver drugs and biomolecules by reducing their toxicity through a simple derivatization process 38. Here, miRNA polyplexes with six different BA-PEIs, including CA, DA, and LA-PEIs, were used at molar feed ratios of 1 and 3 (denoted CA1 for molar feed ratio of 1 and CA3 for a ratio of 3). Each type of BA-PEI and miRNA was complexed based on the method previously described 32, 39, 40 and then characterized (**Table 1**). All miR21 polyplexes formed showed a typical dimension of around 100 nm and a weak positive charge, which was suitable for cellular uptake. The intracellular uptake of Cy5.5-conjugated miR21 polyplexes was examined with confocal laser scanning microscopy and flow cytometry analysis. As shown in **Figure 2A**, the cellular uptake was relatively higher for miR21 complexed with BA-PEI conjugates than for those complexed with BA-unmodified PEI (PEI_1.8_). In particular, CA3, DA3, and LA3 showed better miR21 uptake efficiency than CA1, DA1, and LA1, respectively. The miR21/DA3-treated group showed the strongest fluorescence signal, indicating that DA3-PEI conjugates achieved the highest transfection efficiency. The intracellular delivery of miR21 nanocarriers was further confirmed and quantified with flow cytometry analysis (**Figure 2B**). The cellular uptake was higher for miR21 complexed with CA3, DA3, and LA3 than for that complexed with CA1, DA1, and LA1. Thus, BA-PEI stoichiometric molar feed ratio of 3:1 is more optimum than the molar feed ratio of 1:1 for enhancing membrane permeability. Flow cytometry analysis suggested that the cellular uptake drastically increased with miR21/DA3 treatment as compared to that observed with the control treatment. In particular, 53.7% cells shifted to the second quadrant (R2) in miR21/DA3 treatment group, while only 0.1% and 0.2% of cells shifted to R2 in free miR21 and miR21/PEI_1.8_ groups, respectively.

As confocal microscopy and flow cytometry analysis confirmed the higher transfection efficiency with CA3, DA3, and LA3, we evaluated the cytotoxicity of miR21 nanocarriers using these three types of BA-PEI conjugates (**Figure 2C**). The relative cell viability was remarkably suppressed with CA3 and found to be 0.9 following treatment with miR21/CA3 equivalent to 50 nM miR21 and 0.7 after treatment with miR21/CA3 equivalent to 500 nM miR21. For the groups treated with miR21/DA3 and miR21/LA3, however, no significant decrease in cell viability was observed even at 250 nM miR21 concentration, suggestive of the favorable biocompatibility of miR21 mimic transfection reagent. Based on the results of cellular uptake efficiency and cytotoxicity, DA3 was chosen as the miR21 mimic nanocarrier to perform further *in vitro* and *in vivo* experiments.

### Cytoplasmic delivery of miR21/DA3 and release of miR21 from the polyplexes

**Figure 3:**
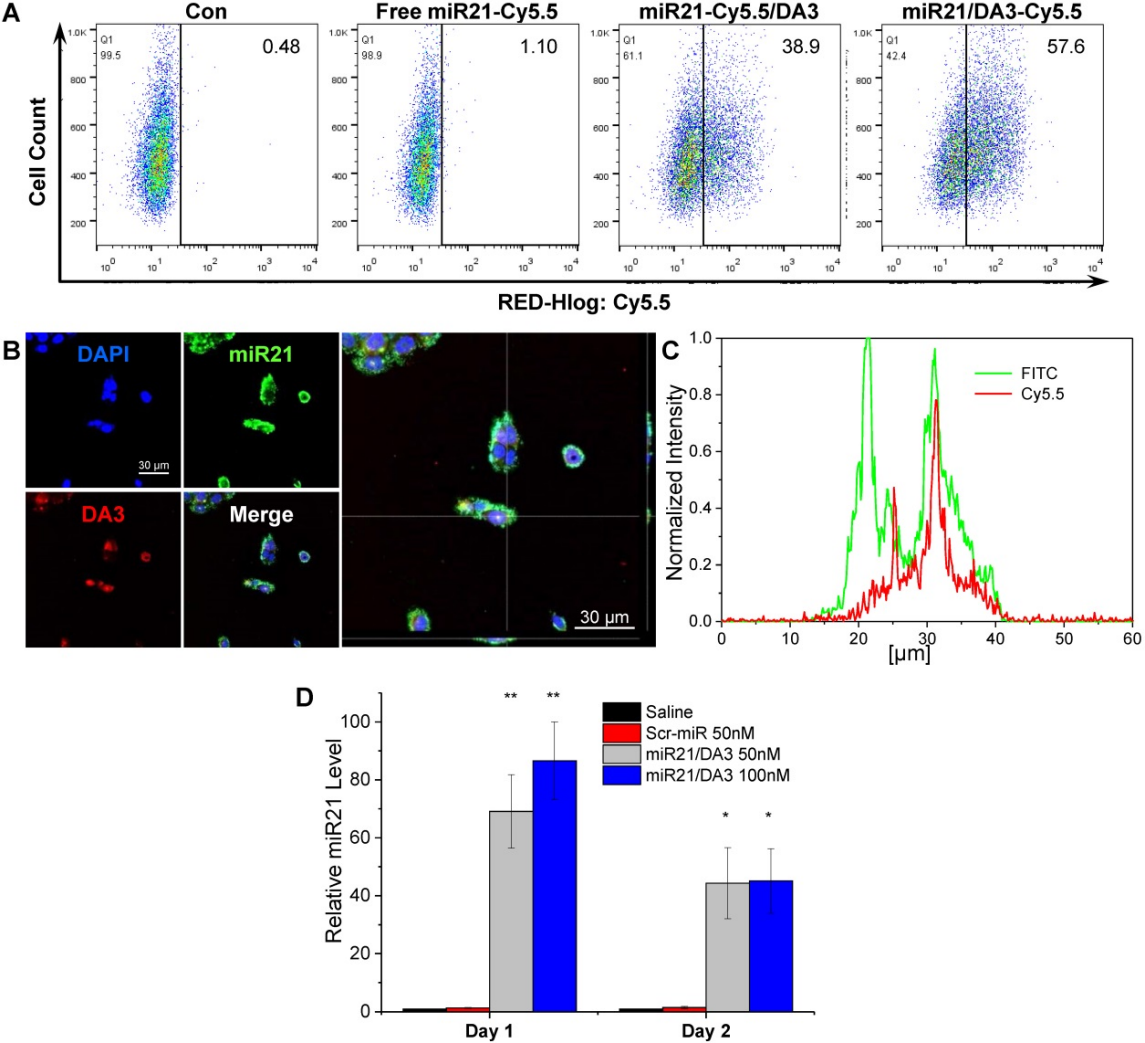
Intracellular delivery of miR21/DA3 and the release of miR21 from polyplexes. (A) Flow cytometry analysis with Cy5.5-labeled miR21/DA3 (miR21-Cy5.5/DA3) and miR21/Cy5.5-labeled DA3 (miR21/DA3-Cy5.5) polyplexes. (B) Confocal fluorescence images showing intracellular release of YOYO1-labeled miR21 from polyplexes. (C) Line scan data of intracellular fluorescence distribution. (D) Relative cytosolic miR21 expression level evaluated with real-time PCR. Data are mean ± *SD* (*n* = 3); *p < 0.05, **p < 0.01 versus saline.

The miRNA-loading efficiency of DA3 polyplexes, as confirmed from gel retardation assay, was about 25% w/w (**Figure S1**). To monitor the cellular uptake and intracellular localization of miRNAs and BA-PEI conjugates, respectively, additional flow cytometry and confocal microscopy analyses were performed using the miR21/DA3 nanocarriers labeled with different fluorescent probes. As shown in **Figure 3A**, both Cy5.5-miR21/DA3 and miR21/DA3-Cy5.5 showed enhanced intracellular uptake of miR21; however, the fluorescence intensity reported for miR21/DA3-Cy5.5-transfected cells was higher than that reported for miR21-Cy5.5/DA3-trasnfected cells; 38.9% and 57.6% cells shifted to the second quadrant for miR21-Cy5.5/DA3 and miR21/DA3-Cy5.5, respectively. This observation may be attributed to the uncomplexed DA3-Cy5.5 remnants adhering to the cell membrane. We obtained z-stack fluorescence images to visualize miR21 released from the polyplexes within the cells (**Figure 3B**). To differentiate miR21 from DA3, we labeled miR21 with YOYO-1 intercalating dye (miR21-YOYO1), while DA3 was labeled with Cy5.5 prior to complexation. After the intracellular delivery of miR21/DA3, the fluorescence signal of DA3 was detected in the restricted regions of the cytoplasm, whereas that of miR21 appeared evenly throughout the cytoplasm. In **Figure 3C**, the z-profiles of normalized fluorescence intensity for a single HaCaT cell confirmed this result, as evident from the stronger and wider fluorescence peaks for miR21 (FITC) than those for DA3 (Cy5.5). These results support the effective release of free miR21 molecules from the polyplexes into the cytoplasm, the site of action for miRNA. In addition, miR21 levels in the cytoplasm after transfection with DA3 were further evaluated with real-time PCR (**Figure 3D**). About 40-fold increase in miR21 expression was observed after treatment with miR21/DA3. In particular, the cells treated with 100 nM miR21/DA3 showed up to 86 times higher miR21 expression than those treated with saline or scrambled miR (Scr-miR) on day 1. On day 2, the relative miR21 level in miR21/DA3-treated cells decreased, indicative of the successful binding of miR21 to the 3'-untsranlated region (UTR) of the target mRNA and its degradation by RNA-induced silence complex (RISC). This observation confirms that miR21 was successfully intracellularly delivered such that it regulated the post-transcriptional expression of genes in the cytoplasm. As strongly cationic gene carriers show poor cytosolic release of nucleic acid molecules even after efficient cellular uptake 41, the miR21/DA3-mediated release of miRNA molecules into the cytoplasm may serve as a favorable strategy to effectively delivery miR21 to skin wounds.

### Activation of the downstream signaling pathway of bone morphogenetic protein (BMP) with miR21/DA3

**Figure 4:**
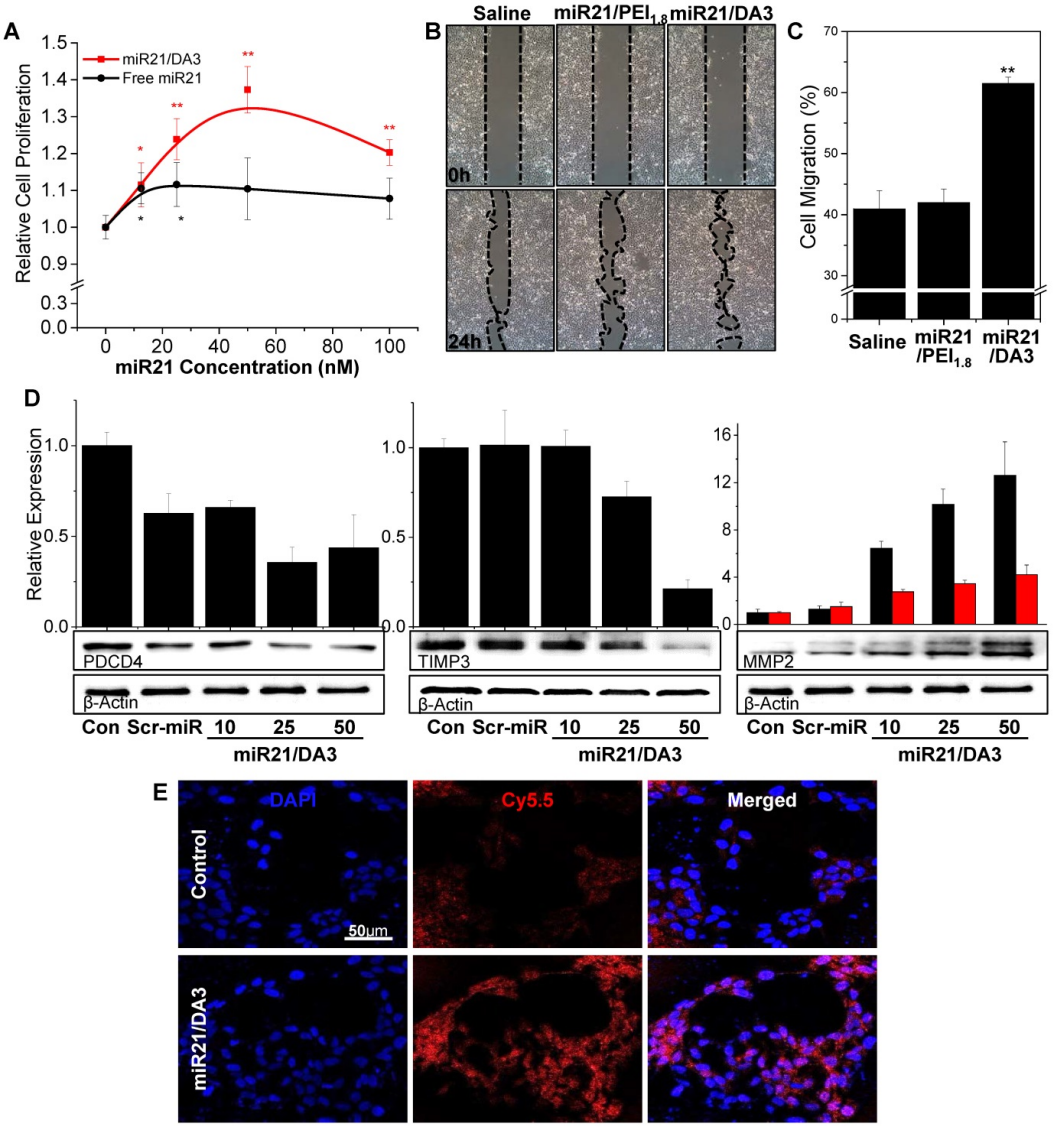
*In vitro* therapeutic effects of miR21/DA3 polyplexes. (A) Cell proliferation assay with free miR21- and miR21/DA3-treated HaCaT cells. (B) Wound scratch migration assay 24 h post-treatment, and (C) quantification of the closed area relative to the scratched area at 0 h. (D) Western blot analysis with HaCaT cells treated with saline, scramble miR/DA3 (Scr-miR), and miR21/DA3, showing PDCD4, TIMP3, and MMP2 expression levels. (E) Real-time MMP2 activity in HaCaT cells visualized with MMP2 probe. Data are mean ± *SD* (*n* = 4); *p < 0.05, **p < 0.01 versus 0 nM miR21 for (A) and **p < 0.01 versus saline for (C).

Keratinocyte proliferation was evaluated in the presence of different concentrations of miR21 (**Figure 4A**). In comparison with free miR21 treatment, miR21/DA3 treatment markedly increased the proliferation of cells. At 50 nM miR21 concentration, the relative cell proliferation was 1.4 and 1.1 for miR21/DA3 and free miR21 group, respectively. We performed wound scratch migration assay to evaluate the effect of miR21/DA3 polyplexes on keratinocyte cell migration. The miR21/DA3-treated group showed remarkably accelerated cell migration as compared to the saline-treated control and miR21/PEI_1.8_ groups; 41%, 43%, and 61% of the scratched area closed after treatment with saline, miR21/PEI_1.8_, and miR21/DA3, respectively (**Figure 4B, C**).

MiR21 is an important downstream component of the BMP signaling pathway and exerts its effects on epidermal keratinocytes by regulating PDCD4 and TIMP3 genes 42. In particular, miR21 downregulates the inhibitory effects of BMP4 on cell proliferation and migration via inhibition of the expression of PDCD4 and TIMP3, which are known to escalate cell proliferation and migration, respectively 25, 43, 44. To examine the molecular mechanisms underlying the role of miR21 mimics in the enhancement of cellular proliferation and migration, PDCD4 and TIMP3 protein expression levels were evaluated with western blot analysis. The treatment with miR21 mimics efficiently suppressed the expression of both PDCD4 and TIMP3 (**Figure 4D**), suggesting that miR21/DA3 could successfully inhibit the downstream signaling pathways of BMP. This effect may be attributed to the improved transfection efficiency with DA3 conjugates. Along with the two target proteins, MMP2 level was also evaluated. MMP2 is a downstream target gene of TIMP3 and directly affects keratinocyte migration and invasion by degrading the extracellular matrix 45, 46. Western blot assays showed that the expression levels of both pro- and active-MMP2 gradually increased with an increase in the concentration of miR21 mimics. For the group treated with 50 nM miR21/DA3, pro- and active-MMP2 levels reached approximately 12- and 4-times the level reported for the control, respectively. In addition, the dynamic MMP2 activity of miR21/DA3-treated cells was visualized with the MMP2 probe developed in our previous research 47. MMP2-activatable probe comprises a Cy5.5-labeled fluorogenic peptide (PLGLR-Cy5.5) and BHQ-3 NIR dark-quencher. Upon exposure of the probe to MMP2, the enzyme cleaves the quencher from the fluorogenic peptide, allowing detection of the peptide fluorescence. Confocal fluorescence images shown in **Figure 4E** reveal the MMP2 activity of HaCaT cells visualized with MMP2 probe. The groups treated with miR21/DA3 showed much stronger fluorescence intensity than those treated with saline after 4 h of probe treatment, indicative of the higher MMP2 level observed following miR21/DA3 treatment. Taken together, *in vitro* experiments demonstrate that miR21/DA3 could effectively suppress BMP signaling pathways through the inhibition of PDCD4 and TIMP3, thereby significantly accelerating the proliferation and migration of keratinocytes.

### *In vivo* effect of miR21/DA3 on wound repair

**Figure 5:**
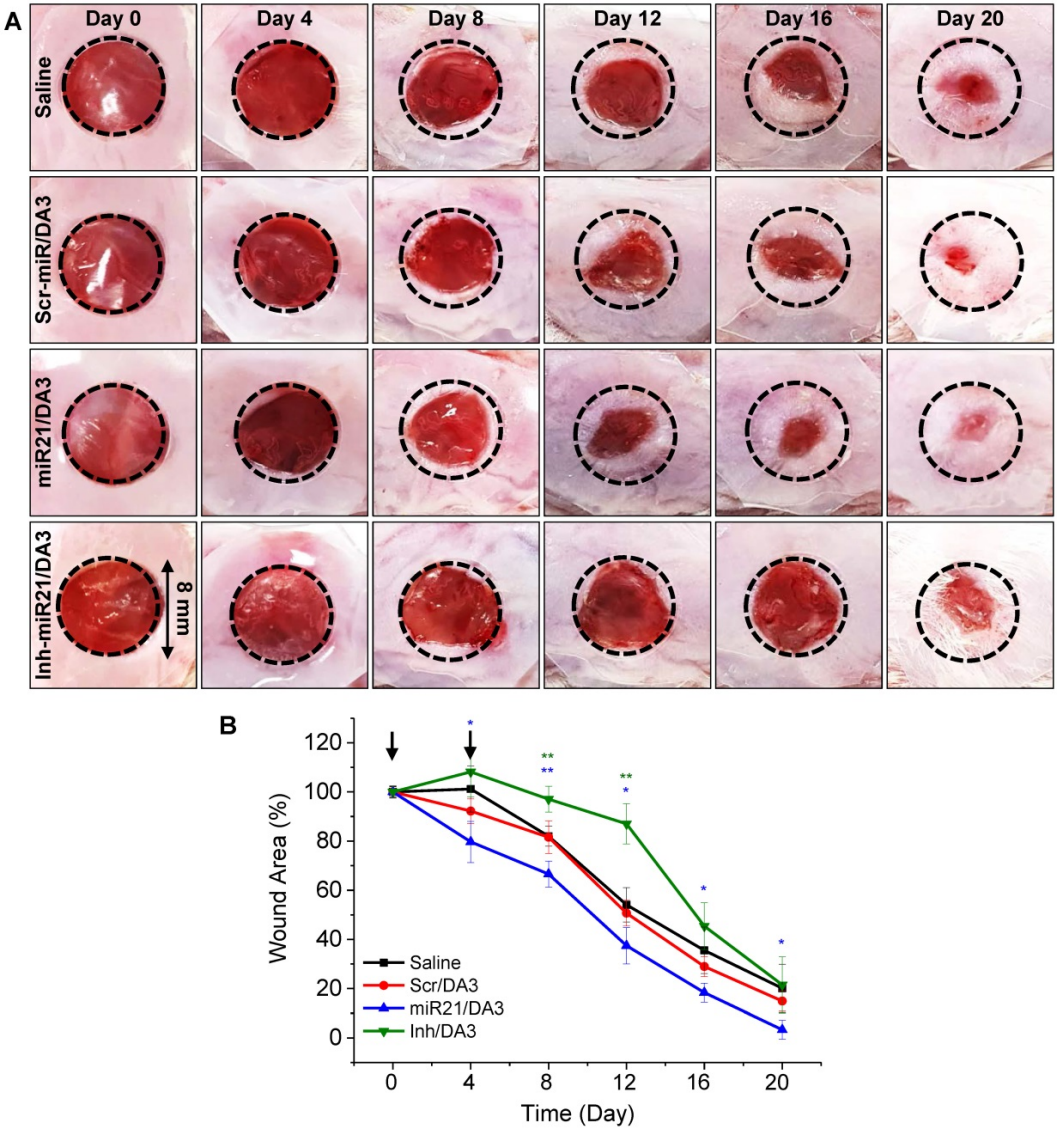
Effects of treatment with miR21/DA3 on cutaneous wound healing. (A) Representative images of wound closure following treatment with 2.5 μM miR21/DA3 on day 0 and 4. A wound was created on the dorsal skin via an 8-mm circular punch biopsy. (B) Quantification of wound size after every 4 days post-wounding. Data are mean ± *SD* (*n* = 5); *p < 0.05, **p < 0.01 versus saline.

To evaluate the effects of miR21/DA3 on wound repair, a mouse excisional wound model was generated using Balb/c mouse. First, the *in vivo* functional specificity of miRNA mimic nanocarriers for wound healing was investigated using an miR21 inhibitor (Inh-miR21) as a negative-control miRNA (**Figure 5**). Only 2.5 μM miRNA/injection was used to minimize the risk of the cytotoxic effects of miR21 nanocarriers. Saline, Scr-miR/DA3, miR21/DA3 and Inh-miR21/DA3 were treated by repetitive administration of half dose of miR21 on day 0 and day 4 post-injury. As shown in **Figure 5A** and** B**, Inh-miR21/DA3 treatment resulted in a significant delay in wound healing as compared to saline and Scr-miR/DA3 treatment, particularly until day 16 post-treatment. Given the artificial elimination of naturally existing miR21, Inh-miR21/DA3-treated negative control group had no choice but to inhibit the normal wound healing process. Thus, wound healing was slower for Inh-miR21 treatment group than for the saline-treated control group. Anti-miR21 was subsequently naturally degraded, which resulted in an increase in the recovery rate at about day 16 post-wounding. On the other hand, miR21/DA3- treated groups showed faster wound healing throughout the experiment.

**Figure 6:**
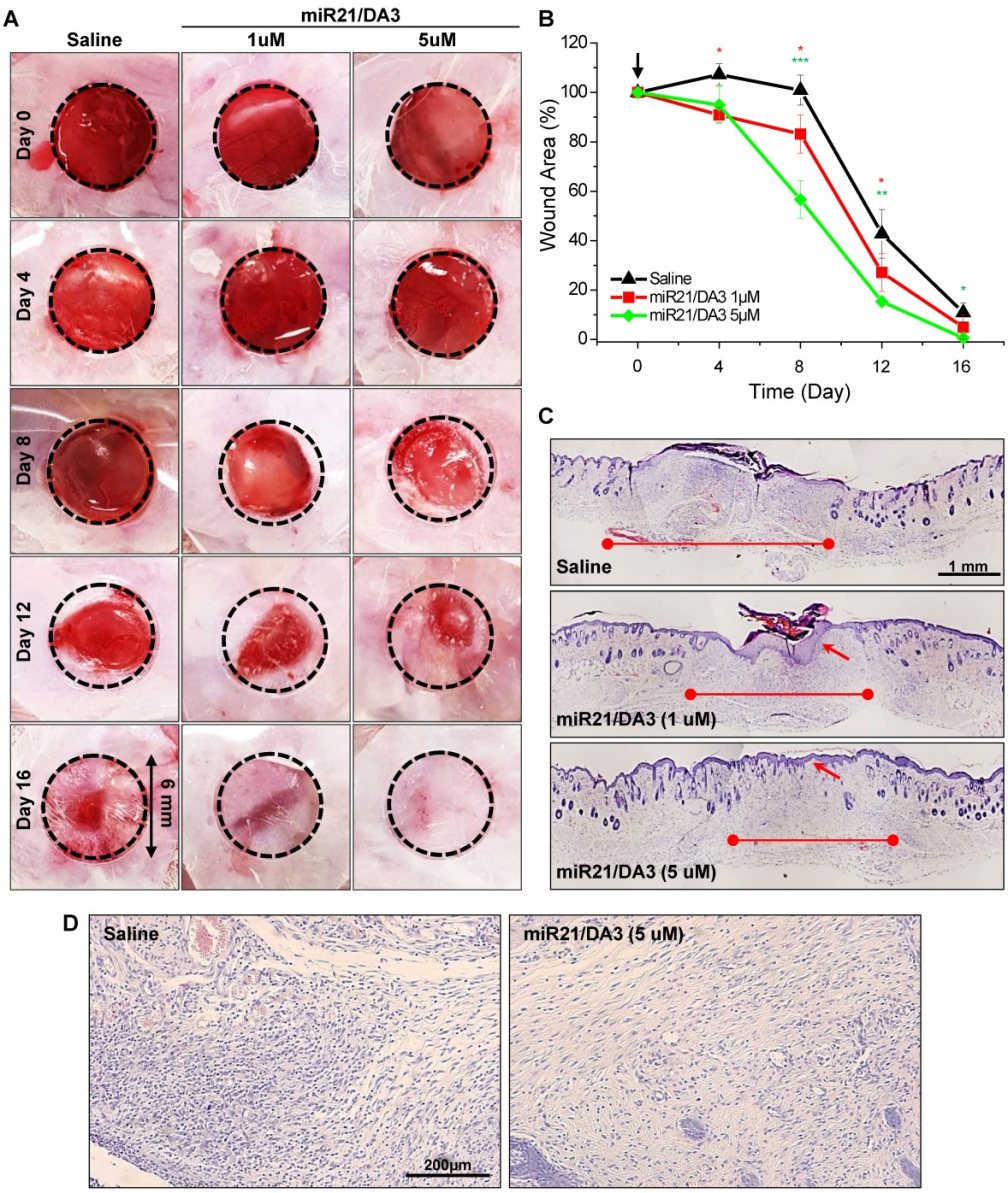
Dose response of miR21/DA3 treatment on cutaneous wound healing. (A) Excisional wounds on the dorsum of Balb/c mice showing wound healing effect of a single treatment with miR21/DA3 *in vivo*. Wound was created on the dorsal skin via a 6-mm circular punch biopsy. (B) Wound area after every 4 days post-wounding. (C) Representative images of whole wound sections at 16 days after injection with saline or 1 and 5 μM of miR21/DA3. (D) Representative magnified photographs of H&E-stained tissue sections at day 16 post-wounding. Data are mean ± *SD* (*n* = 5); *p < 0.05, **p < 0.01, ***p < 0.001 versus saline.

To study miR21 dose response in skin wounds, the cutaneous wounds were examined with a single subcutaneous injection of saline, 1 μM miR21/DA3, or 5 μM miR21/DA3, and monitored until the complete healing of the wound. MiR21 mimics could markedly expedite wound closure in a dose-dependent manner following treatment with miR21/DA3 (**Figure 6A** and** B**). In order to investigate the effects of single therapeutic doses of miR-21/DA3 on wound closure, here we used the mice with an initial wound size, in particular small 6 mm-diameter. After 4 days from treatment, the wounded area was marginally smaller for 5 μM miR21/DA3-treated group than for the control group, but the difference was drastically increased after day 8, as evident from 100% and 57% of open wound observed for the saline- and 5 μM miR21/DA3-treated groups, respectively. Furthermore, the wounds treated with 5 μM miR21/DA3 showed the fastest recovery, with the complete wound closure observed on day 16. Those from the control and 1 μM miR21/DA3-treated groups showed open wounds or scar formation on day 16. In comparison with the saline-treated control group, 1 μM miR21/DA3-treated group showed a slight improvement in the healing effect, especially until 8 days post-treatment. However, the wounds treated with saline healed quickly thereafter, and no statistically significant difference was observed between the saline and 1 μM miR21/DA3-treated groups after day 8. Furthermore, the therapeutic effect of miR21 was confirmed after comparison with nepidermin, which is commonly used as a standard drug for skin wound healing. The wound healing rate with miR21/DA3 was similar to that observed with nepidermin. Co-treatment with miR21/DA3 and nepidermin could further accelerate wound size reduction, particularly during the early stages of wound healing (**Figure S2**). The H&E-stained tissue sections showed a significant acceleration in the process of wound repair following treatment with 5 μM miR21/DA3 as compared to that observed in control and 1 μM miR21/DA3-treated groups (**Figure 6C**). In all miR21/DA3-treated groups, no sign of dermal toxicity was observed. Although 1 μM miR21/DA3 treatment improved tissue granulation and re-epithelization as compared to saline treatment, 5 μM miR21/DA3 treatment expedited the process of wound repair. This was evident from the smallest scar width for this group among the three groups. Moreover, the 5 μM miR21/DA3-treated wounds showed a continuous thin layer of epidermis covering the entire wound bed with hair follicles building underneath. Although 1 μM miR21/DA3-treated wounds showed formation of epidermis, this epidermal layer was much thicker than that observed in the healthy skin tissue adjacent to the edges of wounds, possibly increasing the abnormal scar formation. As shown in **Figure 6D**, the H&E-stained sections of 5 μM miR21/DA3-treated wounds also revealed higher collagen deposition in the dermal layer than that observed in samples from saline-treated control group. In particular, the saline-treated control group showed greater density of epithelial cells rather than collagen deposition, indicative of the ongoing process of epithelialization with fibroblasts and keratinocytes migrating to the wound bed. The group treated with 5 μM miR21/DA3 entered the maturation phase with rearrangement of collagen fibers 48.

### Inhibition of BMP signaling pathways *in vivo*

**Figure 7:**
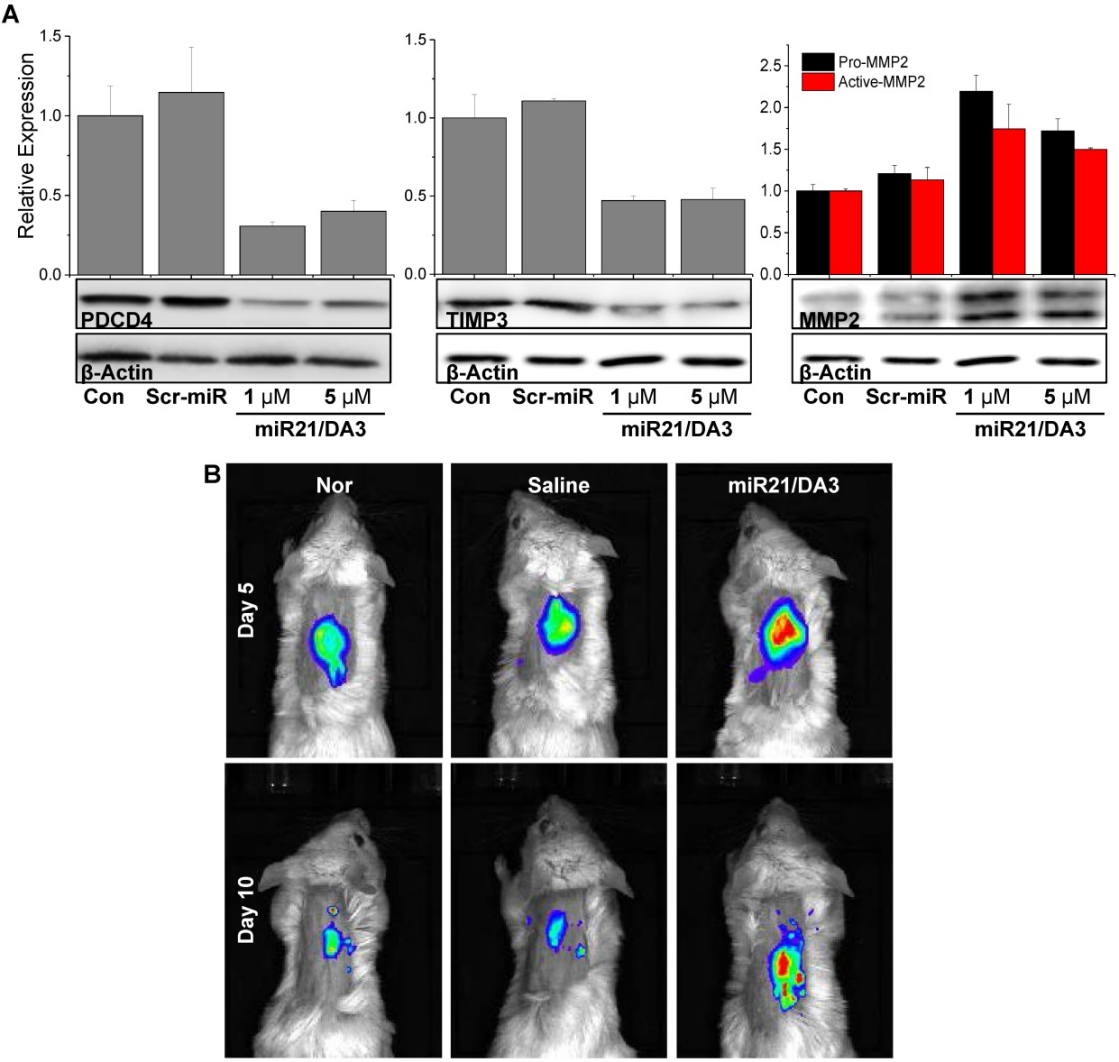
*In vivo* therapeutic effects of miR21/DA3 polyplexes. (A) Western blot analysis with day 5 wound tissue samples showing PDCD4, TIMP3, and MMP2 expression levels. (B) Real-time MMP2 activity was visualized with an MMP2 probe after 5 and 10 days from miR21 treatment.

To examine the signaling pathways involved in miR21 mimic-induced wound healing responses, western blot analysis was performed on wounded tissues on day 5. The protein levels of PDCD4, TIMP3, and MMP2 were assessed, and real-time MMP2 activity was visualized using the MMP2-specific molecular imaging probe. As shown in **Figure 7A**, the expression levels of PDCD4 decreased after miR21/DA3 treatment as compared to that detected in saline-treated and Scr-miR/DA3-treated control groups. Thus, miR21 increased the proliferation of keratinocytes in the wound bed by successfully reducing the level of PDCD4. After the administration of miR21/DA3, the level of TIMP3 drastically reduced that resulted in an increase in the expression of MMP2 by up to two-fold. To visualize local MMP2 activity *in vivo* around the wound bed, the wounds were topically treated with MMP2 probe solutions for overnight after 5 and 10 days from miRNA treatment (**Figure 7B**). While weak fluorescence signals were observed from the normal or wounded mouse skin tissues, the miR21/DA3-treated wounds showed relatively strong fluorescence signals across the wound bed on both days. The overall MMP2 activity around the wound decreased on day 10 due to skin contraction. The elevated levels of MMPs appeared to degrade and loosen the extracellular matrix, thereby allowing local invasion and migration of various surrounding cells, including keratinocytes that are essential for wound re-epithelization 49. Taken together, these results support the effective delivery of miR21 mimics using DA3-PEI-based delivery systems to successfully inhibit BMP signaling via the downregulation of PDCD4 and TIMP3 expression and induction of MMP2 expression, leading to a significant increase in cell proliferation and migration (**Figure 1**).

## Conclusion

In this study, we evaluated miR21 polyplexes complexed with different types of BA-PEI conjugates. BA-PEIs were prepared with CA, DA, and LA as potential carriers for the delivery of miR21 mimic to enhance acute wound healing. Based on the transfection efficiency, cytotoxicity, and effects on cell migration and proliferation, DA3 was chosen as the most optimum carrier for miR21 mimic delivery. The miR21/DA3 polyplexes markedly enhanced the cellular uptake of miR21 mimics and promoted keratinocyte migration and proliferation probably through the successful inhibition of BMP signaling pathways involving PDCD4 and TIMP3, which negatively regulate keratinocyte proliferation and migration. Moreover, the expression level of MMP2 was increased following TIMP3 inhibition, thereby facilitating cell migration through local ECM degradation and softening. The miR21/DA3 nanocarrier system achieved efficient intracellular and transdermal delivery of miR21 mimics and accelerated wound repair *in vivo* through rapid re-epithelialization and better collagen deposition. Therefore, our miRNA nanocarrier system prepared using PEI-DA3 conjugates could be considered as a promising miRNA delivery platform, thereby expanding the use of both synthetic exogenous miRNA mimics and inhibitors to various therapeutic areas for topical treatments.

## Figures and Tables

**Table 1:** Size and surface charge of miR21/BA polyplexes

Carrier	CA1	CA3	DA1	DA3	LA1	LA3	PEI_1.8_
**Size (d.nm)**	120	159	121	173	95.99	181.1	148
**± Dev.**	3.25	5.60	5.30	4.62	4.86	8.43	5.23
**PDI**	0.290	0.277	0.266	0.272	0.222	0.448	0.130
**Charge**	9.2	18.4	23.3	26.7	8.05	21.9	35.6

miR21/BA polyplexes prepared at the BA-PEI polymer/miR21 ratio of 2

## Abbreviations

miRNA: micro ribonucleic acid
TLR-4: Toll-like receptor-4
PDCD4: programmed cell-death protein 4
TIMP3: tissue inhibitors of metalloproteinases 3
TIAM1: T cell lymphoma invasion and metastasis 1
BA-PEI: bile acid-conjugated polyethyleneimine
CA: cholic acid
DA: deoxycholic acid
LA: lithocholic acid
VEGF: vascular endothelial growth factor
MMP2: matrix metalloproteinase 2
siRNA: small-interfering ribonucleic acid
PEI1.8: 1.8 kDa low molecular weight PEI
miR21/DA3: miR21 complexed with DA3-PEI conjugates
BMP: bone morphogenetic protein
Scr-miR/DA3: scramble miRNA complexed with DA3-PEI conjugates
Inh-miR21/DA3: miR21 inhibitor complexed with DA3-PEI conjugates
rhEGF: recombinant human epidermal growth factor
