# Supplementary material for: Development of microRNA-21 mimic nanocarriers for the treatment of cutaneous wounds
Source: Theranostics. 2020 Feb 10;10(7):3240–53. doi: 10.7150/thno.39870 (PMC7053209; doi:10.7150/thno.39870)
Supplement: Supplementary file 1 — Supplementary figures. [file thnov10p3240s1.pdf]

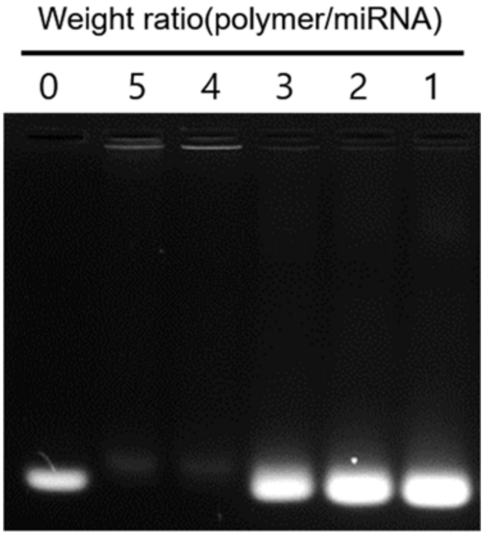

Figure S1. Gel retardation assay of miR21/DA3 nanocomplexes. Equal amounts of DA-PEI were incubated with various concentrations of miRNAs for 30 min.

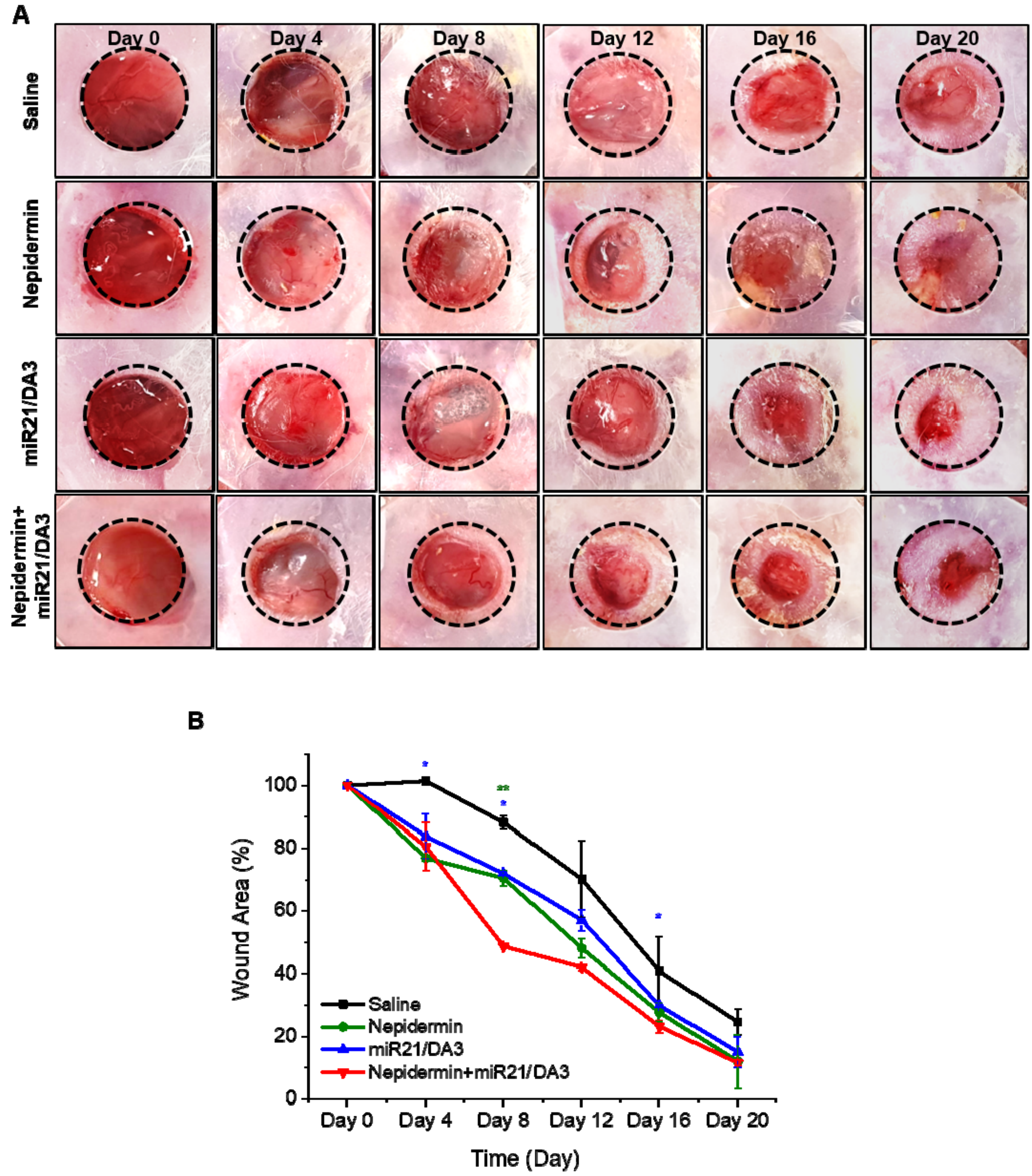

Figure S2. Effects of miR21/DA3 and Nepidermin treatment on cutaneous wound healing. (A) Representative images of wound closure following treatment with 2.5 μM miR21/DA3 and 0.5 g of Nepidermin on day 0. Wound was created on the dorsal skin via an 8-mm circular punch biopsy. (B) Quantification of the wound size after every 4 days post-wounding. Data are expressed as mean ± SD (n = 3); *$p < 0.05$, **$p < 0.01$ versus saline.
